# Retraction: MiR-30e-5p inhibits the migration and invasion of nasopharyngeal carcinoma via regulating the expression of MTA1

**DOI:** 10.1042/BSR-2019-4309_RET

**Published:** 2023-02-10

**Keywords:** Invasion, Migration, MiR-30e-5p, MTA1, Nasopharyngeal carcinoma

This Retraction follows an Expression of Concern relating to this article previously published by Portland Press. This article is being retracted from *Bioscience Reports* at the request of the Editor-in-Chief and the Editorial Board following receipt of a notification from a reader, alerting the Editorial Board to concerns regarding the Kaplan-Meier graphs of Figure 1F and G, as well as the western blots. The graphs appear to show an implausible date range, and the western blots have concerning features such as the shapes of the bands and absence of any detectable background. The authors have been contacted with regards to the retraction and have not responded to the Journal's queries or the concerns raised. Given the extent of the issues raised, the Editorial Board stand by the decision to retract the article.

